# *Leishmania* Manipulation of Sand Fly Feeding Behavior Results in Enhanced Transmission

**DOI:** 10.1371/journal.ppat.0030091

**Published:** 2007-06-29

**Authors:** Matthew E Rogers, Paul A Bates

**Affiliations:** Liverpool School of Tropical Medicine, Liverpool, United Kingdom; National Institute of Allergy and Infectious Diseases, United States of America

## Abstract

In nature the prevalence of *Leishmania* infection in whole sand fly populations can be very low (<0.1%), even in areas of endemicity and high transmission. It has long since been assumed that the protozoan parasite *Leishmania* can manipulate the feeding behavior of its sand fly vector, thus enhancing transmission efficiency, but neither the way in which it does so nor the mechanisms behind such manipulation have been described. A key feature of parasite development in the sand fly gut is the secretion of a gel-like plug composed of filamentous proteophosphoglycan. Using both experimental and natural parasite–sand fly combinations we show that secretion of this gel is accompanied by differentiation of mammal-infective transmission stages. Further, *Leishmania* infection specifically causes an increase in vector biting persistence on mice (re-feeding after interruption) and also promotes feeding on multiple hosts. Both of these aspects of vector behavior were found to be finely tuned to the differentiation of parasite transmission stages in the sand fly gut. By experimentally accelerating the development rate of the parasites, we showed that *Leishmania* can optimize its transmission by inducing increased biting persistence only when infective stages are present. This crucial adaptive manipulation resulted in enhanced infection of experimental hosts. Thus, we demonstrate that behavioral manipulation of the infected vector provides a selective advantage to the parasite by significantly increasing transmission.

## Author Summary

Leishmaniasis is a human parasitic disease that can result in destructive skin lesions or sometimes in fatal visceral involvement if left untreated. Infections are acquired via the bites of female sand flies, small blood-feeding insects. In this experimental study using mice, we show that *Leishmania* parasites can manipulate the behavior of sand flies so that infected flies become more tenacious, returning to feed if interrupted more readily and delivering more bites than uninfected flies. This change in behavior occurs only when the parasites have become infective to humans and are ready for transmission. At the same time, the parasites in the sand fly secrete a viscous gel that blocks up the gut and mouthparts and interferes with feeding. The result of this manipulation is an increase in the severity of disease and number of infections. These findings help us to better understand how human infection occurs, which is important when trying to devise ways of controlling this disease. It is currently estimated that at least 12 million people have leishmaniasis, with 2 million new infections every year.

## Introduction

Parasites exhibit myriad adaptations to ensure their survival and transmission from host to host. These include manipulation, where infection elicits a specific behavioral response from the host of selective benefit to the parasite [1–3]. For vector-borne diseases like leishmaniasis there are two hosts to consider: the invertebrate host and the mammalian host. These come together at a critical point in the parasite life cycle, transmission. Any manipulation by the parasite that increases both the contact with and biting of the mammalian host will combine to increase the probability of transmission. The ability of parasites (nematodes, protozoa, bacteria, or viruses) to influence the feeding of their arthropod vectors has long been observed [2–4]; however, in the majority of examples, neither the manipulator molecules involved nor the effect on transmission, has been demonstrated.

The protozoan *Leishmania* is a parasite of both humans and animals transmitted by the bite of female phlebotomine sand flies. The World Health Organization has estimated that 12 million people are infected with *Leishmania* and 350 million are at risk of infection [5]. However, despite the medical importance of this tropical parasitic disease, surprisingly little is known of the interaction between *Leishmania* parasites and the feeding behavior of the sand fly vector. After being ingested by the sand fly along with the blood, *Leishmania* amastigotes transform to motile promastigote forms and undergo a complex development, limited to the gut of the sand fly [6]. In all *Leishmania* species, the final phase of development results in the colonization of the junction between the sand fly anterior midgut and foregut at the stomodeal valve, a structure that regulates blood intake during feeding. It is here that the parasites undergo their final multiplication and differentiation into highly motile mammal-infective metacyclic promastigote forms [6], a process termed metacyclogenesis.

Recent work on transmission of leishmaniasis demonstrated that metacyclic promastigotes of Leishmania mexicana are regurgitated from the midgut of the sand fly vector accompanied by a viscous gel-like material of parasite origin [7]. This promastigote secretory gel (PSG) is a potent parasite virulence factor, and, together with sand fly saliva, significantly enhances cutaneous infections when co-delivered into the skin of the mammalian host [7,8]. The major component of the gel responsible for disease exacerbation is filamentous proteophosphoglycan (fPPG), a very high molecular mass glycoprotein unique to *Leishmania* [9].

In its quest for transmission, *Leishmania* can also considerably modify the sand fly gut environment by damaging the stomodeal valve [10,11] and physically blocking the gut with fPPG, which forms the 3-D matrix of the parasite-derived gel [7,12]. The combination of these two events results in the blockage of the anterior midgut with a plug of *Leishmania* promastigotes and their gel, which distends and permanently holds open the already eroded valve. This is hypothesized to adapt the sand fly for transmission by promoting the reflux of both parasites and gel during blood feeding, dubbed the “blocked fly hypothesis” [13]. The resulting blockage of sand flies, first noted in the early twentieth century [14], interferes with feeding and limits the volume of blood a fly can obtain [15–17]; and, probably as a consequence, infected flies have been noted to probe the skin more frequently and spend more time feeding [15–18]. Although the observation that infected sand flies can experience difficulty with feeding is not new, the connection between this behavior and the likelihood of increased parasite transmission was not made until the late 1970s when Killick-Kendrick and co-workers [16] re-analyzed the data of Chung et al. [15]. They showed that Phlebotomus chinensis were more likely to transmit Leishmania donovani to hamsters when flies probed and took no blood compared to those infected flies that took a blood meal. This demonstrated that *Leishmania* could manipulate the feeding ability of the sand fly to promote its own transmission success; however, neither the extent of the manipulation nor the mechanisms that underlie it have been investigated since.

These various previously described effects of the parasite on the sand fly host promote host-vector contact and transmission, and are thus potentially advantageous to the parasite, but are useful by-products of infection rather than being examples of true manipulation. Moreover, it is important to bear in mind that host contact and blood feeding do not come without risks and present a considerable danger to both vector and parasites alike when the host defends itself [19], so it is not clear if any of these effects are selectively advantageous. In such a scenario, the timing of transmission opportunities is critical in order to minimize the risk of being killed and to promote the survival of the vector long enough to complete a parasite's development to infectiousness [1,2,19,20]. In this study, we investigate the behavior of *Leishmania*-infected sand flies. We describe an elegant and novel example of behavioral manipulation in which the timing of parasite development is linked to the feeding persistence of the sand fly vector and demonstrate that such a strategy enhances *Leishmania* transmission.

## Results/Discussion

### *Leishmania* Acquire Their Infectivity during the Formation of the Gel Plug

**Figure 1:**
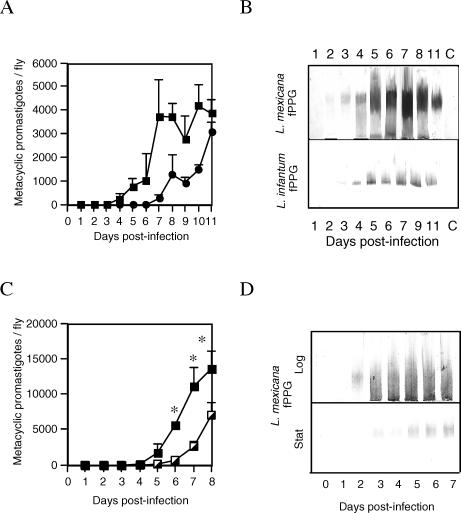
Metacyclogenesis and fPPG Synthesis in L. mexicana and L. infantum (A) Flies were infected by membrane feeding with amastigotes of L. mexicana (filled squares) or L. infantum (filled circles), ten flies/group harvested daily, and the average number of metacyclics/fly determined. Error bars represent 1 s.e.m. (B) Accumulation of fPPG within the midguts of infected sand flies. Midgut homogenates from flies infected with L. mexicana (upper panel) or L. infantum analyzed by SDS-PAGE and immunoblotting using AP3 mAb to detect the presence of fPPG glycans. Lane C is a day 7 uninfected blood-fed control sample. (C) Metacyclogenesis in sand flies infected with L. mexicana exponential phase– (closed squares) or stationary phase– (half squares) cultured amastigotes. Error bars represent 1 s.e.m. Asterisks indicate statistically significant differences (*p* < 0.05) from stationary infections using an unpaired *t*-test. (D) PSG accumulation in sand flies infected with L. mexicana exponential phase (upper panel) or stationary phase (lower panel) amastigotes analyzed by SDS-PAGE and immunoblotting using AP3.

The two known parasite-mediated events that directly enhance transmission of *Leishmania* are metacyclogenesis and secretion of fPPG. Since either or both could exert some pathogenic and/or manipulative effect on the sand fly host, we investigated their kinetics in detail. Sand fly infections initiated with tissue-derived amastigotes of both L. mexicana (experimental combination) and Leishmania infantum (natural combination) in Lutzomyia longipalpis were produced, and the generation of metacyclic promastigotes and fPPG synthesis was examined (Figure 1). The onset of metacyclogenesis was more rapid and the numbers of metacyclic promastigotes higher in L. mexicana than in L. infantum (Figure 1A). This was mirrored in the quantity and appearance of fPPG (Figure 1B), being first detected on day 2 versus day 4 in *L. mexicana–* and L. infantum–infected flies, respectively. To test this apparent linkage further, the rate of development was deliberately modulated by infecting flies with L. mexicana axenic amastigotes in either exponential or stationary growth phase [21]. Amastigotes in either growth status can transform to and grow as promastigotes, but those in exponential growth phase accomplish this transition more quickly. Infection of flies with exponential phase axenic amastigotes accelerated transformation and differentiation compared to infection with stationary phase amastigotes, causing both metacyclic promastigotes (Figure 1C) and fPPG (Figure 1D) to appear earlier and in greater amounts. Thus, these data demonstrate a direct association between metacyclogenesis and fPPG secretion.

### *Leishmania* Reduce the Lifespan of Their Vectors

**Figure 2:**
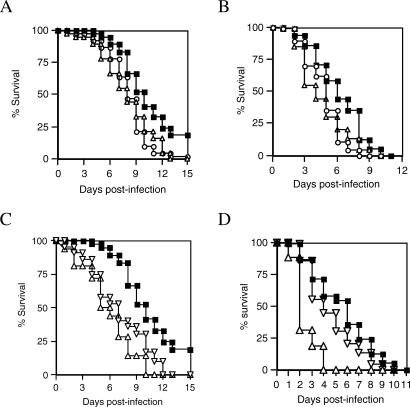
Kaplan–Meier Plots Showing Survival of Lutzomyia longipalpis Under Various Experimental Conditions. (A) Normal insectary conditions. Flies were fed by membrane feeding on rabbit blood alone (filled squares), blood containing L. mexicana lesion amastigotes (open triangle), or L. infantum splenic amastigotes (open circle). Groups of 300 fed female flies were monitored daily for the occurrence of new deaths. (B) Stress conditions. The experiment was performed as in (A), except that flies were stressed on a daily basis by incubating them at 16 °C without a sugar meal for 2 h and forcing them to fly during a 30-min period by continuously agitating the cage. (C) Effect of L. mexicana developmental rate on survival of sand fly vector. Flies were infected with L. mexicana exponential phase– (up-ended open triangle) or stationary phase– (down-ended open triangle) cultured amastigotes and maintained under normal insectary conditions. (D) The experiment was performed as in (C), except that flies were stressed on a daily basis as described for (A).

The development of transmissible *Leishmania* infections in the sand fly gut seems likely to exact a fitness cost on the fly host. For example, the considerable distension of the gut by the gel-like plug [17], damage to the stomodeal valve [10,11], and the diversion of nutrient resources to parasite growth [22] each have potential for causing pathology to sand flies. We examined the effect of infection on the longevity of *Lu. longipalpis.* Both L. mexicana and L. infantum infections were found to significantly reduce longevity under normal lab conditions (log rank test: *p* < 0.0001), from a median of 11 d for controls to 9 d for both L. mexicana– and L. infantum–infected flies. Although little is known regarding the survival of flies in the wild, in some respects the natural environment may be more stressful and hazardous than the conditions experienced in laboratory insectaries. Therefore, the survival of flies exposed to a combination of stress conditions was also investigated (Figure 2B). Again, infections with L. infantum or L. mexicana were both found to reduce sand fly longevity compared to controls. Stress conditions by themselves reduced the longevity of uninfected control flies from a median of 11 d to 7 d, but caused an even more marked reduction in both groups of infected flies than under benign conditions (L. mexicana normal versus stressed: median longevity 9 d versus 5 d, *p* < 0.0001; L. infantum normal versus stressed: median longevity 9 d versus 6 d, *p* < 0.0001). A final test of longevity was made by infecting flies with either exponential phase– or stationary phase–cultured L. mexicana amastigotes (Figure 2C and 2D). Flies with accelerated *Leishmania* development derived from exponential phase amastigotes experienced significantly earlier mortality than those infected with stationary phase amastigotes, and the pathogenicity of these infections was exacerbated when stress conditions were applied (logarithmic versus stationary, normal conditions: median longevity 5 d versus 7 d, *p* < 0.0001; logarithmic versus stationary, stressed conditions: median longevity 3 d versus 5 d, *p* < 0.0001). The conclusion of these experiments is that infection of *Lu. longipalpis* with *Leishmania* exerts a fitness cost as expressed by reduced longevity, and that this is linked to parasite developmental rate.

### *Leishmania* Do Not Alter Sand Fly Fecundity

Next we examined possible effects of *Leishmania* on fecundity, since infection of certain vectors (for example, malaria in mosquitoes) can reduce reproductive fitness by causing production of fewer eggs [2]. In mosquitoes, it has been suggested that this may be a compensatory mechanism to ameliorate reduced longevity by re-diverting resources that would otherwise be used for egg production and egg laying, enabling the parasite to complete development to infective sporozoite forms. However, when we examined this possibility, none of the *Leishmania* infections described here (L. mexicana/*L. infantum,* stressed or unstressed) had any significant impact on immediate reproductive fitness as measured by egg production (Table S1), indicating that this is not a strategy used by *Leishmania* in this vector.

### Hypothesis: *Leishmania*-Infected Sand Flies Are More Persistent in Blood Feeding

The data described above and presented in Figures 1 and 2 indicate that an interesting balance exists in the *Leishmania*–sand fly relationship: more aggressive and quickly developing infections will lead to increased numbers of metacyclics and fPPG (e.g., Figure 1C and 1D), thereby increasing the probability of transmission; but this will also exert a greater fitness cost on the vector (e.g., Figure 2C), thereby reducing the probability of transmission. Thus, although parasite developmental rate does vary between parasite–vector combinations and can also be influenced by experimental or environmental conditions in the laboratory and field, a priori there is no obvious selective benefit to the parasite of developing more quickly or more slowly. In the face of these potentially conflicting influences on transmission, any alteration of vector feeding behavior by the parasite that will independently increase the probability of transmission will be of clear selective advantage. Such behavior might include factors encouraging a fly to find a host and begin feeding and those that encourage a fly to continue to feed and/or re-feed. Also the defensive behavior of the mammalian host itself is important, as this will influence the success of vector blood feeding [23]. Therefore, the timing of vector feeding with respect to parasite development is a crucial behavior, since successful transmission of parasites requires that their vectors survive at least long enough for the parasites to complete their development to an infective stage (the extrinsic incubation period). In the rodent malaria–mosquito model of Plasmodium yoelii in *Anopheles stephensi,* infected mosquitoes experienced an increase in feeding persistence once the extrinsic incubation period was completed [20]. Feeding persistence is the repetition of feeding attempts when prevented from feeding, i.e., a behavioral mechanism in the face of simulated host defensive behavior. Such biting persistence of a vector to resume feeding after interruption is considered an important parameter of vectorial capacity, which theoretically could promote transmission to multiple hosts [24]. Therefore, we investigated whether infection of *Lu. longipalpis* increased their feeding persistence.

### *Leishmania* Manipulates Sand Flies to Persist with Blood Feeding

**Figure 3:**
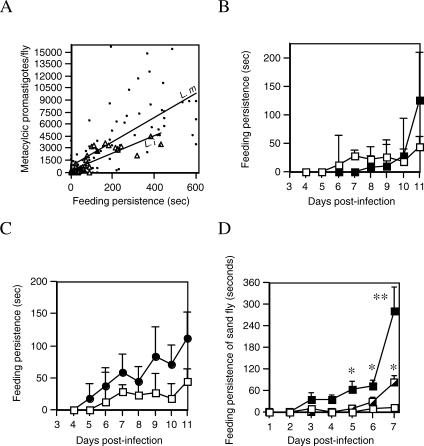
Feeding Persistence of Sand Flies (A) Relationship between numbers of metacyclic promastigotes in *Lu. longipalpis* and feeding persistence. Flies were infected with L. mexicana (closed circle) or L. infantum (open triangle) amastigotes. From 4 d post-infection flies were assayed for their feeding persistence in a 10-min behavioral assay and then dissected to determine the total number of metacyclic promastigotes present. The feeding persistence of each individual fly is plotted against the number of metacyclic promastigotes they harbored. Linear correlation coefficient, *r* ^2^ = 0.504 for L. mexicana (L. m), and 0.705 for L. infantum (L. i). (B) Feeding persistence of L. infantum–infected flies fed rabbit blood alone (open square) or infected with L. infantum amastigotes (closed square). The persistence of flies exposed individually to an anaesthetized mouse was observed daily. *n* = 16 sand flies per point, representing the combination of two independent experiments. Error bars 1 s.e.m. (C) Feeding persistence of L. mexicana–infected flies, experimental design as in (B). Flies were fed rabbit blood alone (open square) or infected with L. mexicana (closed circle). (D) Effect of *Leishmania* development on sand fly feeding persistence, experimental design as in (B). Flies were infected with exponential (closed square) or stationary (half square) phase L. mexicana-cultured amastigotes. Asterisks indicate values from exponential and stationary infections that are statistically significant (*p < 0.05, **p < 0.005) from blood-fed control flies using an unpaired *t*-test.

Control or infected flies were offered an anaesthetized mouse but were prevented from continued feeding by gentle brushing of the antennae after probing. When the assay was complete, each fly was dissected to determine the number of metacyclic promastigotes present. The results showed that both L. mexicana– and L. infantum–infected flies displayed a positive correlation between persistence and number of metacyclic promastigotes/fly (Figure 3A). When the timing of persistence was examined in L. infantum–infected sand flies there was no increase apparent in the early phase of infection, up to day 9, but thereafter a significant increase compared to control flies (Figure 3B). The increase in feeding persistence during days 10–11 occurred concurrently with the increase in metacyclic promastigotes in L. infantum–infected flies (Figure 1A). A similar result was observed in L. mexicana–infected flies, and crucially the increase in persistence was apparent earlier (Figure 3C) as was the appearance of metacyclic promastigotes (Figure 1A). Thus, there was a strong correlation between increased feeding persistence and the accumulation of infective forms. To directly test this association, we again used L. mexicana axenic amastigotes to modulate parasite developmental rate. Sand flies infected with exponential phase amastigotes showed an early onset of increased persistence compared to those infected with stationary phase amastigotes (Figure 3D). In these experiments, the exponential phase amastigote infections increased the persistence of the sand fly 22-fold and 7-fold on day 7 compared to uninfected control and stationary phase amastigote–infected flies, respectively (average persistence ± SE: blood control, 13 s ± 11 s; stationary-*L. mexicana,* 89 s ± 20 s; exponential-*L. mexicana,* 290 s ± 72 s). These results demonstrated for the first time that *Leishmania* can control this critical aspect of sand fly feeding behavior.

We also determined the duration and frequency of feeding attempts in uninterrupted sand flies. With regard to duration, flies with mature L. infantum and L. mexicana infections (i.e., with metacyclic promastigotes and fPPG in their anterior midgut) took an average of 1.3 and 2.4 times longer (total time) to feed on anaesthetized mice compared to uninfected flies (average feeding time ± SE of blood control, 322 s ± 40 s; L. infantum–infected, 419 s ± 44 s; *L. mexicana*–infected, 759 s ± 52 s), and in accordance with the blocked-fly hypothesis, these infected flies were more likely to obtain only a partial meal of blood (Tables S2–S4). Both of these results confirm the reduced feeding capacity of infective sand flies. With regard to frequency of biting, a possible consequence of an insufficient blood meal is that this would encourage a fly to attempt to feed more frequently. However, interestingly, when this was tested we found that infected flies exposed to unconscious mice did not demonstrate increased probing; rather, they remained feeding for longer until either they finally engorged (often partially) or they gave up completely. This shows that both the presence of an infection in the fly and the interruption of biting are required to reveal the observed alterations to vector feeding behavior. This makes sense, because under natural conditions potential hosts are obviously not anesthetized and sand flies can give a noticeable, even painful bite, so host defensive behavior is very likely in such cases. Further, even where the bite is not noticeable interruption could easily occur through the normal activity of the host disturbing the feeding activity of the sand fly.

### *Leishmania*-Infected Sand Flies Feed on Multiple Hosts

**Figure 4:**
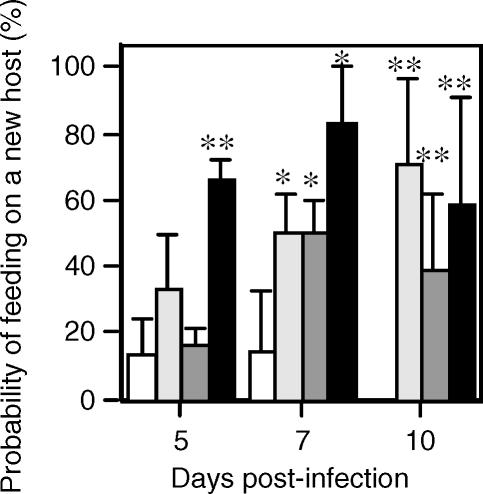
Feeding Persistence and Transmission of *Leishmania* to Multiple Hosts Flies were fed blood alone (open square), L. infantum (dotted square), L. mexicana stationary phase (shaded square), or exponential phase amastigotes (closed square). The number of flies feeding on a different host when interrupted was determined on days 5, 7, and 10 post-infection/feeding in a behavioral assay. *n* = 12 per point, representing the combination of two independent experiments. Error bars 1 s.e.m. Asterisks indicate values that are statistically significant (*p < 0.05, **p < 0.005) from blood fed control flies using an unpaired *t*-test.

Importantly, for the increase in feeding persistence of infected flies to be of adaptive value to the parasite the behavior must lead to increased transmission. During a transmission attempt interruption to blood feeding can arise either from an insufficient blood flow to the bite site or from the activation of host defensive behavior [2,23]. In both scenarios a partial blood meal is likely to promote a fly to re-feed in order to obtain sufficient nutrients for egg-maturation, and as indicated above, persistence is further enhanced in the case of a *Leishmania*-infected sand fly. Although re-feeding on the same host would clearly benefit the parasite, re-feeding on multiple hosts is particularly advantageous as this will increase the basic reproduction number (R_0_) of the infection (the number of new cases of a disease that arises from a single infection). Such behavior has been proposed with malaria-infected mosquitoes [24], but there is only circumstantial evidence for this to apply to *Leishmania*-infected sand flies [25,26]. Therefore, we modified the interruption-behavioral assay by including a second anaesthetized mouse in the cage to assess the probability of the fly choosing to feed on a different host when interrupted (Figure 4). Flies were tested at 5, 7, and 10 d post-infection to provide infections at different stages of metacyclogenesis (beginning, middle, and end) for both *Leishmania* species. The results showed that infection with both L. infantum and L. mexicana promoted feeding on multiple hosts, whereas uninfected flies in most cases gave up completely when interrupted. Furthermore, this behavior correlated with metacyclogenesis, because exponential phase L. mexicana infections exhibited an increased and earlier tendency to initiate a feed on a new host earlier than infections initiated with stationary phase L. mexicana amastigotes (*p* < 0.005). These data demonstrated that persistent feeding behavior can lead to an increase in the number of hosts that become infected.

### *Leishmania* Manipulation of Sand Fly Behavior Increases the Infective Inoculum

**Figure 5:**
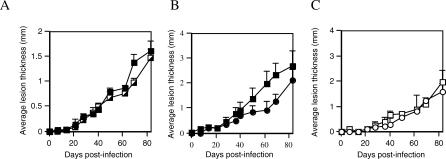
Feeding Persistence and Infectivity (A) Infectivity of fly-derived L. mexicana metacyclic promastigotes. Flies were infected with exponential phase or stationary phase L. mexicana-cultured amastigotes, infections allowed to mature (day 7), then metacyclic promastigotes were obtained, washed, and 10^3^ parasites injected into the right foot of each BALB/c mouse. Data from two groups of six mice are shown, infected with metacyclic promastigotes from exponential phase– (closed square) or stationary phase– (half-square) derived infections. (B and C) Influence of feeding persistence on *Leishmania* infection in mice. Flies were infected with exponential (closed square or open square) or stationary phase (closed circle or open circle) L. mexicana-cultured amastigotes. Day 7–infected flies were exposed individually to the right paw of anaesthetized BALB/c mice and allowed to feed for 15 min with ([B], closed circle or closed square) or without ([C], open square or open circle) interruption.

The final issue we considered was whether this behavior could also lead to an increased number of parasites per infected host. As a preliminary experiment, we first investigated whether there was any inherent difference in infectivity of metacyclic promastigotes obtained from exponential phase or stationary phase–initiated fly infections (Figure 5A). These populations proved to be of equal infectivity when isolated from flies and needle-injected into BALB/c mice; therefore, any difference in outcome cannot be ascribed to a difference in the quality of the parasites. To assess the direct consequence of vector behavioral manipulation for *Leishmania* transmission and infection we repeated the persistence assay with individual exponential and stationary phase L. mexicana amastigote–infected flies on individual BALB/c mice; however, in this experiment the mice were maintained to monitor the evolution of the resulting cutaneous lesions (Figure 5B). Day 7–infected flies were used because of the large differences in feeding persistence demonstrated in previous experiments (Figure 3D). This experiment revealed a significant difference in lesion pathology between the two groups of mice (*p* < 0.05); the more persistent exponential phase–infected flies generating more aggressive infections (average ± SE fly persistence: exponential = 193 s ± 94 s; stationary = 35 s ± 16 s). This was also reflected in the final parasite burdens for the two groups of mice (3.72 ± 0.64 ×10^8^ versus 2.28 ± 0.82 ×10^8^ amastigotes/lesion). From the above we know this difference was due to the delivery of parasites by the sand fly and not from any qualitative differences in parasite infectivity (Figure 5A). In a parallel experiment where infected flies were allowed uninterrupted feeding on mice, the courses of the resulting infections were not significantly different (Figure 5C), and the accompanying parasite burdens were very similar. This is an important control because it shows that the infections in the two populations of flies are equally capable of infecting mice, but what is different is the persistence of the flies in the face of interruption (Figure 5B). Collectively, these data showed for the first time that persistent vector feeding behavior can lead to both an increase in the numbers of parasites transmitted per host and the number of hosts infected.

### Conclusions

Is this an example of adaptive or nonadaptive manipulation of vector behavior? It is notoriously difficult to dissect these apart; however, the current data satisfy the criteria described by Poulin [27] and Thomas et al. [1], enabling us to conclude that the behavioral manipulation is adaptive. First, it is complex—the manipulation relies on the successful establishment and development of the parasite within the sand fly and this requires many intrinsic barriers to be overcome [6]. Second, it shows evidence of “purposive design”—the behavior is exhibited when the parasite is ready for transmission. Third, the fPPG gel appears to have arisen independently in other *Leishmania*–sand fly combinations [12] and has interesting similarities with the polysaccharide biofilm production and mechanism of Yersinia pestis (plague) transmission by the flea [28,29]. Fourth, and most importantly, we have shown that the manipulation directly increased the fitness of the parasite through enhanced transmission. This manipulation was shown to correlate closely with metacyclogenesis of the parasites and the accumulation of the fPPG gel that accompanies this process. fPPG is a potent virulence factor for *Leishmania* that benefits both the transmission of parasite from the sand fly and infection of the mammalian host [7].

Previously, it was suggested that the attachment of *Leishmania* parasites in contact with the foregut might interfere with the function of mechanoreceptors that detect blood flow, and this might explain the reduced ability of flies to take a blood meal [4,30]. However, in view of the results presented here, we propose that the functioning of these mechanoreceptors is impaired by fPPG, which plays the role of manipulator molecule. This in turn may promote the hunger state and the persistence of the fly, or alternatively, increase the threshold blood volume at which blood-seeking behavior is inhibited. Currently, we are applying a genomics approach to investigate the effect of infection on sand flies [31], which may enable us to identify genes involved in the manipulation. Further, we ultimately aim to examine the role of manipulation in a field setting.

In this study, we report that under experimental conditions *Leishmania* parasites can manipulate the feeding behavior of the sand fly to promote its own transmission. Our data reveal that under conditions of interruption, the feeding persistence of the infected sand fly increased in parallel with the development of the parasite, the number of transmission stages (metacyclic promastigotes), and the accumulation of fPPG. As a consequence, flies with the highest number of metacyclic promastigotes were also found to be more likely to initiate a second feed on naïve hosts, and the lesions generated from the bites of individual infected flies demonstrated that this form of behavioral manipulation directly results in enhanced parasite transmission. This study shows that *Leishmania* transmission is the product of the following: the physical blockage of the gut with fPPG that ensures regurgitation of infective forms [7,12,13]; the subsequent exacerbation of infection in the mammalian host through the action of fPPG and vector saliva [7,8]; and now the manipulation of feeding behavior according to the presence of infective forms available for transmission. These factors highlight the close coevolution between the *Leishmania* parasite and its sand fly host. This study significantly improves our understanding of *Leishmania* transmission, and these findings can now be incorporated into both epidemiological and experimental models of infection.

## Materials and Methods

### *Leishmania* culture and morphology.

L. mexicana (MNYC/BZ/62/M379) or L. infantum (syn. Leishmania chagasi) (MHOM/BR/76/M4192) were cultured as previously described [22,32]. Formalin-fixed samples of parasites were counted using a Neubauer hemocyctometer, and their developmental morphology assessed through measurement of parasites from Giemsa-stained smears, using the system of Rogers et al. [17].

### Sand fly infection and survival.

Five-day-old Lutzomyia longipalpis (Jacobina strain) female sand flies were infected with L. mexicana or L. infantum amastigotes through an artificial membrane feeding system at a density of 2 × 10^6^ amastigotes/ml in fresh rabbit blood [33]. L. mexicana amastigotes were harvested from the rump lesions of female BALB/c mice, and L. infantum amastigotes were isolated from the spleens of BALB/c mice. In some experiments, L. mexicana amastigotes were axenically cultured prior to use [34]. Blood-fed flies were separated and maintained under a 12-h light:dark cycle at 28–30 °C, 80%–95% relative humidity, and supplied with 70% (w/v) sucrose ad libitum. Flies were denied the opportunity to lay eggs to minimize post-oviposition mortality [16], and all dissected flies with mature infections were observed to contain eggs (gravid). In some experiments, female flies were stressed on a daily basis by incubating them at 16 °C without a sugar meal for 2 h, and by forcing them to fly during a 30-min period by agitating the cage. To assess sand fly survival, cages were monitored daily for the occurrence of new deaths. Statistical analysis of survival data was performed using the log rank test [35].

### PSG accumulation.

Pooled infected sand fly homogenates were immunoblotted to detect fPPG using monoclonal antibody AP3 as previously described [7].

### Biting persistence assay.

The method of Anderson et al. was used [20] with slight modifications. Feeding persistence was defined as the amount of time (up to a maximum of 10 min) that a sand fly continued to land and insert their mouthparts after being interrupted. BALB/c mice that were age and weight matched were used as the source of the blood meal in each experiment. The mice were anesthetized and placed into a 25-cm^3^ netted cage; their bodies were screened with netting except for their right leg, to introduce an element of searching during feeding. Care was taken to keep the same orientation and position of the mice for each exposure. Sand flies were released into the cage singly, and the time taken for each fly to find the right paw and insert their mouthparts for a feed was recorded. A sand fly was removed and recorded as a “no-feed” when 10 min after release had elapsed without it initiating a feed. Flies that began feeding within this time were allowed 1 min before interruption by gently brushing the antennae. The disturbance was repeated, allowing 10 s of feeding before the next disturbance, until the sand fly gave up or took longer than 1 min before re-feeding. The total time a fly spent attempting to re-feed was recorded as the measure of feeding persistence. Following each observation, sand flies were transferred to a glass vial, knocked down on ice, and midguts were dissected, homogenized, and the parasites quantified as described [17].

### Second host choice assay.

A modified form of the persistence assay was used, in which flies were assayed individually for their decision to feed on an alternative host in the face of interruption. Two anesthetized BALB/c mice were kept 10 cm apart in a 25-cm^3^ cage and exposed to single flies. When a fly began feeding on a mouse it was allowed a further minute before it was interrupted. Following this, the fly was interrupted every 10 s if it tried to remain feeding on the same mouse. Feeding interruption was carried out until either the fly initiated a feed on the opposite mouse, or until 15 min elapsed. Flies were prevented from feeding by gentle brushing of the antennae between feeding attempts. Flies were dissected after the assay to confirm infection where appropriate.

### Infection of mice.

Female BALB/c mice were infected either by injection of 10^3^ metacyclic promastigotes via needle into the dorsal surface of the right hind foot or by allowing single infected sand flies to bite at the same site [7]. Lesion development was monitored by measuring the swelling of the right foot with Vernier callipers and subtracting the width of the contralateral uninfected foot. At the end of experiments, mice were humanely euthanized, and parasite burdens in the feet determined either by direct counting via hemocytometer or by limiting dilution. All procedures involving animals were approved by a local Animal Welfare Committee and performed in accordance with United Kingdom Government (Home Office) and EC regulations. Sequential measurements for each individual mouse were used to calculate the area under the curve for a plot of lesion thickness against time [36]. The distribution of values did not show evidence of non-normality using the Shapiro-Wilk test and therefore parametric analysis was performed (*t* tests). The null hypothesis was rejected if *p* < 0.05. Statistical analysis was performed using the StatsDirect software package version 2.3.1.

## Supporting Information

Table S1 Effect of *Leishmania* Infection and Environmental Stress Conditions on Sand Fly Fecundity A total of 50 female flies per group were dissected to determine the number of eggs they produced 4 d following infection or blood feeding. A further total of 50 female flies per group were allowed to lay eggs in oviposition pots for 7 d at which time the number of flies surviving were recorded and removed. The laid eggs were counted and maintained in the oviposition pots for a further 7 d when the number of larvae that hatched was determined. The results represent the combination of two independent experiments. No significant differences in fecundity were found between groups.
(42 KB DOC)

Table S2 Blood Meal Volume Obtained by Uninfected Sand Flies Five-day-old flies were exposed individually to a single anaesthetized mouse and the relative size of their blood meal obtained (none, partial, or full) after one feeding attempt was recorded upon dissection.
(37 KB DOC)

Table S3 Effect of L. mexicana Infection on Blood Meal Volume Obtained by Sand Flies All flies were infected with 2 × 10^6^ lesion amastigotes per ml rabbit blood by membrane feeding. Flies with 4- to 10-d-old infections were exposed individually to a single anaesthetized mouse and the relative size of their blood meal obtained (none, partial, or full) after one feeding attempt was recorded upon dissection, together with the total number of parasites within each fly. The data are pooled from multiple experiments.
(38 KB DOC)

Table S4 Effect of L. infantum Infection on Blood Meal Volume Obtained by Sand Flies All flies were infected with 2 × 10^6^ splenic amastigotes per ml rabbit blood by membrane feeding. Flies with 4- to 10-d-old infections were exposed individually to a single anaesthetized mouse and the relative size of their blood meal obtained (none, partial, or full) after one feeding attempt was recorded upon dissection, together with the total number of parasites within each fly. The data are pooled from multiple experiments.
(38 KB DOC)

## Abbreviations

fPPG: filamentous proteophosphoglycan
PSG: promastigote secretory gel
