# Supplementary material for: Leishmania Manipulation of Sand Fly Feeding Behavior Results in Enhanced Transmission
Source: PLoS Pathog. 2007 Jun 29;3(6):e91. doi: 10.1371/journal.ppat.0030091 (PMC1904410; doi:10.1371/journal.ppat.0030091)
Supplement: Table S1 — A total of 50 female flies per group were dissected to determine the number of eggs they produced 4 d following infection or blood feeding. A further total of 50 female flies per group were allowed to lay eggs in oviposition pots for 7 d at which time the number of flies surviving were recorded and removed. The laid eggs were counted and maintained in the oviposition pots for a further 7 d when the number of larvae that hatched was determined. The results represent the combination of two independent experiments. No significant differences in fecundity were found between groups. (42 KB DOC) [file ppat.0030091.st001.doc]

**Table S1**

| Infection type | Stressed/Unstressed | Eggs per fly  (mean±SD) | Eggs laid per fly  (mean) | % larvae hatched | % flies surviving oviposition |
| --- | --- | --- | --- | --- | --- |
| Uninfected | Unstressed | 73±12 | 41 | 38 | 43 |
| Uninfected | Stressed | 72±15 | 23 | 62 | 10 |
| *L. mexicana* | Unstressed | 75±12 | 27 | 56 | 19 |
| *L. mexicana* | Stressed | 55±15 | 18 | 56 | 0 |
| *L. infantum* | Unstressed | 62±12 | 30 | 51 | 32 |
| *L. infantum* | Stressed | 48±19 | 22 | 40 | 20 |
