# Supplementary material for: Leishmania Manipulation of Sand Fly Feeding Behavior Results in Enhanced Transmission
Source: PLoS Pathog. 2007 Jun 29;3(6):e91. doi: 10.1371/journal.ppat.0030091 (PMC1904410; doi:10.1371/journal.ppat.0030091)
Supplement: Table S2 — Five-day-old flies were exposed individually to a single anaesthetized mouse and the relative size of their blood meal obtained (none, partial, or full) after one feeding attempt was recorded upon dissection. (37 KB DOC) [file ppat.0030091.st002.doc]

**Table S2**

| Meal type | % flies |
| --- | --- |
| No meal (n=0) | 0 |
| Full meal (n=29) | 97 |
| Partial meal (n=1) | 3 |
| Combined (n=30) | 100 |
