# Supplementary material for: Leishmania Manipulation of Sand Fly Feeding Behavior Results in Enhanced Transmission
Source: PLoS Pathog. 2007 Jun 29;3(6):e91. doi: 10.1371/journal.ppat.0030091 (PMC1904410; doi:10.1371/journal.ppat.0030091)
Supplement: Table S4 — All flies were infected with 2 × 10^6^ splenic amastigotes per ml rabbit blood by membrane feeding. Flies with 4- to 10-d-old infections were exposed individually to a single anaesthetized mouse and the relative size of their blood meal obtained (none, partial, or full) after one feeding attempt was recorded upon dissection, together with the total number of parasites within each fly. The data are pooled from multiple experiments. (38 KB DOC) [file ppat.0030091.st004.doc]

**Table S4**

| Meal type | % flies | Average infection size per fly  (mean±SE) |
| --- | --- | --- |
| No meal (n=0) | 0 | - |
| Full meal (n=17) | 61 | 1.2x10^4^±7.22x10^3^ |
| Partial meal (n=11) | 39 | 1.82x10^4^±6.43x10^3^ |
| Combined (n=28) | 100 | 1.64x10^4^±4.47x10^3^ |
